# Tracking variations in daily questionable health behaviors and their psychological roots: a preregistered experience sampling study

**DOI:** 10.1038/s41598-023-41243-w

**Published:** 2023-08-28

**Authors:** L. B. Lazarević, G. Knežević, D. Purić, P. Teovanović, M. B. Petrović, M. Ninković, M. Živanović, S. Stanković, M. Branković, P. Lukić, G. Opačić, I. Žeželj

**Affiliations:** 1 Faculty of Philosophy, Institute of Psychology, University of Belgrade, Belgrade, Serbia; 2 Faculty of Philosophy, Laboratory for Research of Individual Differences, University of Belgrade, Belgrade, Serbia; 3 Faculty of Philosophy, Department of Psychology, University of Belgrade, Čika Ljubina 18-20, 11000 Belgrade, Serbia; 4 Faculty of Special Education and Rehabilitation, University of Belgrade, Belgrade, Serbia; 5 Institute for Philosophy and Social Theory, University of Belgrade, Belgrade, Serbia; 6 Faculty of Media and Communication, Singidunum University, Belgrade, Serbia

**Keywords:** Psychology, Human behaviour, Health policy

## Abstract

People resort to various questionable health practices to preserve or regain health - they intentionally do not adhere to medical recommendations (e.g. self-medicate or modify the prescribed therapies; iNAR), or use traditional/complementary/alternative (TCAM) medicine. As retrospective reports overestimate adherence and suffer from recall and desirability bias, we tracked the variations in daily questionable health behaviors and compared them to their retrospectively reported lifetime use. We also preregistered and explored their relations to a wide set of psychological predictors - distal (personality traits and basic thinking dispositions) and proximal (different unfounded beliefs and biases grouped under the term irrational mindset). A community sample (N = 224) tracked daily engagement in iNAR and TCAM use for 14 days, resulting in 3136 data points. We observed a high rate of questionable health practices over the 14 days; daily engagement rates roughly corresponded to lifetime ones. Both iNAR and TCAM were weakly, but robustly positively related. Independent of the assessment method, an irrational mindset was the most important predictor of TCAM use. For iNAR, however, psychological predictors emerged as relevant only when assessed retrospectively. Our study offers insight into questionable health behaviors from both a within and between-person perspective and highlights the importance of their psychological roots.

## Introduction

People use various medical practices in an effort to maintain or recover their health. While some of them are advised by health authorities and health providers, people sometimes simply do not follow their advice and/or engage in practices outside of the official medicine. In addition to decreasing the effectiveness of treatments for acute and chronic diseases^1^ and, consequently, decreasing patients’ quality of life, non-adherence also burdens the healthcare system^2^. Psychologically, unintentional non-adherence which can be due to a lack of socio-economic or cognitive resources is not as relevant as intentional non-adherence (iNAR), i.e. a patient deciding to ignore or modify medical advice either by changing the dosage or length of medication, avoiding scheduled check-ups or deciding to self-medicate^3^. A previous study showed that participants, on average, intentionally decided not to follow almost half of the medical recommendations at some point in their lifetime^3^.

The World Health Organization uses the term ‘traditional, complementary, and alternative medicine (TCAM)’ to merge health practices outside official medicine^4^. While it sometimes can be beneficial, TCAM is, despite popular opinion, not harmless: it can lead to adverse effects, delay conventional treatment, interact with it unexpectedly, or divert people from it altogether (e.g.^5^). Nevertheless, TCAM remains popular among people, and its use is constantly growing^6^. Apart from the widespread belief in its safety, the popularity of TCAM is also due to beliefs in its naturalness, as well as, in the case of traditional medicine, its historical and cultural roots^7^.

In this paper, we track these two types of questionable health behaviors daily for fourteen days, relate them to retrospective reports of the same behaviors, and explore their psychological roots.

### Roots of questionable health behaviors

People engage in questionable health behaviors due to a variety of reasons, from system-related to person-related ones. In this study, we focus on person-related predictors of iNAR and TCAM use and divide them into three conceptual categories.

The first category consists of the obvious candidates - an individual’s socio-demographic background and health status. Namely, there is empirical support for TCAM use being associated with female sex, younger age, higher education, and higher socio-economic status (for a review, see Ref.^8^). Less adherence to treatment and more frequent TCAM use was also observed in people with poorer health, especially those suffering from chronic disease^9, 10^.

Our target behaviors seem, however, to also be psychologically more deeply rooted. There is ample evidence that individual differences in questionable health practices can be traced back to differences in personality traits and basic thinking dispositions. For example, Big Five low Conscientiousness appeared to be the most reliable predictor of long-term medication non-adherence, while low Agreeableness and high Neuroticism only sometimes played a role (e.g. Ref.^11^). On the other hand, high Openness was robustly associated with TCAM use (for a review, see Ref.^12^). Recent studies that focused on non-adherence to COVID-19-related health recommendations^13, 14^, emphasized the importance of personality traits outside of the Big Five model - low Honesty from the HEXACO model^15^ and higher proneness to psychotic-like experiences and behaviors (i.e. Disintegration^16, 17^). As for thinking dispositions, TCAM use and preference were typically related to a less pronounced rational thinking style^18^, and lower cognitive reflection^19^. While there is no direct evidence of the role of thinking styles in non-adherence to medical recommendations, there are studies relating more intuitive, experiential style to more negative attitudes toward evidence-based practices^20^.

In addition to these basic traits, certain beliefs and biases are more directly related to questionable health behaviors. We group them under the umbrella term irrational mindset and define it as a set of beliefs that do not adhere to the standards of normative logic, lack an evidence base, and persist even when confronted with disconfirming evidence. Recent studies demonstrated that TCAM use was predicted by magical health beliefs^21^, superstition^22^, proneness to medical conspiracies^23^, and general conspiracist beliefs^24^, as well as the simultaneous endorsement of contradictory beliefs (i.e. doublethink^25^). TCAM use was also consistently related to susceptibility to naturalness bias^10, 26^, illusory correlations^10^, belief bias, and, more generally, probabilistic reasoning biases^27, 28^. Although there is less evidence, there are indications that an irrational mindset can also be predictive of certain aspects of non-adherence to medical recommendations, especially intentional non-adherence. Self-medicating, changing the prescribed treatment, and avoiding checkups were shown to be positively, albeit weakly related to conspiratorial thinking and superstitious beliefs^3^. Similarly, people more prone to medical conspiracy theories more often avoided health and dental checkups^23^. During the COVID-19 pandemic, non-adhering to public health guidelines was once again related to conspiratorial thinking, but also to overconfidence bias^28^. In an attempt to track down a tendency that accounts for beliefs and biases in an irrational mindset, some authors^29^ name illusory pattern perception, or apophenia, an automatic proneness to perceive the relation between unrelated phenomena^30^. To our best knowledge, there are no studies relating apophenia with questionable health behaviors. To broaden the irrational mindset, we added a new category to it - personal irrational beliefs, labeled as such because they are inflexible, unrealistic, and harmful to individuals. These beliefs pertain to individuals’ views of themselves, others, and the world, and have been linked to various negative life outcomes, but research on their impact on health behaviors remains limited (for the initial evidence of their relevance see Ref.^14^).

To get a better overview of important psychological predictors, we decided to complement the set of irrational beliefs with a group of socio-political beliefs, indicative of a person’s relation to their social and political environment, for which there is evidence they can be related to health behaviors. For example, trust in the healthcare system and physicians has been repeatedly shown to be an important predictor of normative health behaviors, such as patients’ adherence to treatment (see Ref.^31^ for a review). Furthermore, trust in science has been shown to positively predict recommended health behaviors, such as vaccination, whilst it negatively predicted the use of pseudoscientific health practices^26^. Religiosity and spirituality were found to be robustly related to TCAM use (e.g. Ref.^32^) and sometimes to non-adherence as well^33^. There is also scarce evidence suggesting political orientation predicts adherence to medical recommendations, mainly in the domain of public health. Right-wing political orientation has been linked to higher vaccine hesitancy both before (e.g. Ref.^34^) and during the COVID-19 pandemic (e.g. Ref.^35^), as well as to more frequent use of alternative remedies, such as homeopathy and essential oils^36^.

### Experience sampling method in tracking health-related behaviors

Most of the studies tracking questionable health behaviors rely on retrospective self-reports^37^. Existing data suggest that they tend to overestimate adherence and are prone to recall bias, social desirability bias, and errors in self-observation^37, 38^. Stemming from the problems with such measures, there is a call for more reliable behavioral assessment, which relies on ambulatory assessment, and specifically the experience sampling methodology (ESM^39^). In ESM studies, indicators of behaviors, cognitions, or emotions are collected momentarily, in real-time, and during regular daily activities. Participants are prompted to respond to repeated assessments, once or several times per day. This enables more reliable, ecologically valid, and structured data collection. There are important advantages of ESM compared to traditional assessment methods: they are more robust to typical sources of errors emerging in self-report assessment^40^, and they enable more direct and valid insight into the phenomena. In addition, they offer a prospective, within-person approach, which contrasts with the between-person approach of the retrospective reports^41^.

A limited number of health-behavior studies that did use ESM, mostly focused on patients with chronic conditions, like chronic pain^42^, hypertension (e.g. Ref.^43^), or psychopathology (e.g. Ref.^44^), limiting the applicability of conclusions and recommendations outside the populations studied. Additionally, these studies mostly focus on medication adherence, neglecting other non-adherence behaviors of clinical importance, such as self-medication or going to doctor appointments (what we labeled iNAR). Maybe even more importantly, despite all the stated advantages of the method, to our knowledge, there are no studies tracking daily TCAM behaviors. The design that combines two assessment methods would also allow for testing the robustness of different predictors of retrospectively and daily assessed questionable health behaviors.

### The current study

So far, studies typically focused on irrational beliefs from a single domain and one type of health behavior, mostly TCAM practices. Personality traits, on the other hand, were studied in relation to specific forms of non-adherence, mostly to medications prescribed for specific health conditions and not for intentional non-adherence to recommendations. Although the existing evidence suggests these psychological variables should be studied as predictors of iNAR and TCAM use, their predictiveness has never been tested in a comprehensive design. In the current study, we investigated the relationship between a large set of interconnected irrational thinking tendencies and beliefs, basic personality traits and thinking dispositions on one hand, and two types of questionable health practices on the other.

We used ESM to track daily TCAM usage and iNAR behaviors in a sample drawn from the general population and related these behaviors to a wide set of psychological predictors. Our main objectives were: (a) to explore the frequency of TCAM and iNAR behaviors over a 14-day period in a community sample and: (b) to test the predictive validity of a wide spectrum of person-centered variables for both daily variations and retrospectively measured TCAM and iNAR behaviors. We grouped the psychological predictors into distal (personality traits and basic thinking dispositions) and proximal ones (irrational mindset and socio-political beliefs) and tested their predictivity over and above sociodemographics and health-related variables. Additionally, to gain more insight into the quality of methods for tracking the behaviors, we compared TCAM and iNAR measured daily and their retrospective self-report.

### Hypotheses

We preregistered eight core hypotheses (https://osf.io/4vxdk/?view_only=45fde338501b4b1f801a072a347f479c), which we outline following our grouping of variables into (1) socio-economic and health-related, (2) distal, and (3) proximal predictors of TCAM and iNAR. When articulating the hypotheses, we relied on robust empirical evidence for retrospectively assessed health behaviors, as there were almost no studies with the daily tracked data. This way we also tested whether the same patterns of relations were to be observed irrespectively of the assessment method.

We expected women to be more prone to use TCAM (H1) (e.g. Ref.^14, 45^), while we did not have any specific expectations regarding other socio-demographic variables. As for the distal predictors, we expected Disintegration (H2a^13^) and experiential thinking style (H2b) to be positively related to TCAM^18, 19^. On the other hand, the existing evidence shows that analytical thinking style (H3a^18^) and cognitive reflection (H3b) should be negatively related to TCAM^19–28^. We expected that irrational beliefs, specifically medical conspiracies (H4a^23^), magical beliefs about health (H4b^21^), and naturalness bias (H4c^10^), would positively predict TCAM use. We also expected that more content-specific predictors (e.g. magical beliefs about health, and belief in medical conspiracy theories) will emerge as the best predictors of TCAM (as observed in^10^) (H5). We expected the relation of iNAR to irrational beliefs to be positive as well, but weaker compared to the relation of the TCAM-irrational beliefs (H6^3, 10^). Finally, we expected iNAR to correlate negatively with trust in healthcare professionals and positively with mistrust in the healthcare system (H7; e.g. Ref.^3, 46^), and TCAM use to be more pronounced in more religious individuals (H8^47^).

In the exploratory portion of the analyses, we tested how TCAM use and iNAR relate to other indicators of an irrational mindset, namely, superstition, personal irrational beliefs, proneness to doublethink, and a number of cognitive biases (overconfidence, commitment, and belief bias, illusory correlation, and biases in probability reasoning).

## Method

### Disclosures

All data are available on the Open Science Framework project page (https://osf.io/4dq9m/?view_only=c11adfeea7af4d93af0e147399aaeafd). We report how we determined our sample size, all data exclusions, all manipulations, and all measures in the study. All participants signed informed consent and voluntarily participated in the study. All procedures adhered to the principles of the Declaration of Helsinki.

### Sample

We calculated that a sample of at least *N* = 191 would enable the detection of correlations ≥ 0.20, with desired power of 0.80, if the alpha level is set at 0.05. This number of entities at level two is far larger than the number found to lead to biased estimates of the second-level standard errors in multi-level random coefficient modeling (meaning a sample of 50 or less^48^).

Initially, 300 respondents were recruited for the retrospective assessment phase. Only participants who completed the retrospective assessment and passed all three embedded attention checks (N = 268) were invited to take part in the experience sampling study. The final sample - those who fully completed both phases - consisted of 224 participants with an average age of 41.5 years (*SD* = 12.6) and 53.6% female. The dropout rate was 15%, similar to what is reported in the meta-analysis by^49^. Two-thirds of the sample came from urban (66.5%) areas. Half of the sample (55.3%) completed college or university, 43.3% completed high school, and only 1.3% completed elementary school. A total of 62.5% were married or living with a partner, 7.1% were divorced, and 30.4% reported they were single.

### Procedure

The Ethics committee of the Faculty of Philosophy, University of Belgrade, approved the study (#935/1), available at https://osf.io/bv7yh. Participants were recruited by a professional research agency (IPSOS Serbia) and received a gift voucher (equivalent to 10 EUR) as compensation. Retrospective measures were administered first via the online platform SoSci Survey^50^. Next, we collected daily assessments of questionable health behaviors using xSample—A free-to-use Android OS and iOS supported application, developed to apply experience sampling methodology^51^. ESM assessment comprised the two questionnaires designed for the study to track daily TCAM and iNAR behaviors. Participants received standard push notifications once per day, at 7 PM, as a reminder, and could respond to the survey between 7 PM and 11 PM. They filled in the survey every day for 14 days (not necessarily consecutive), during the 28-day time window. The application is programmed to stop sending notifications when all assessment points are collected. Half of the sample (49%) finished the study in 14 consecutive days, while an additional 43% completed it in up to 21 days. The longest period to finish the study was 27 days (*N* = 2).

### Missing data

To be included in the analysis, participants needed to have completed both the retrospective measures and the 14 ESM recordings. There were no missing data in either the retrospective or ESM part of the study since responding was set as mandatory. In total, we collected 3136 ESM recordings (data points).

### Variables and instruments

#### Questionable health behaviors - criterion variables

iNAR-12^3^ is a 12-item self-report checklist assessing intentional non-adherence to official medical recommendations. Participants are presented with a list of situations reflecting non-adherence and asked to respond to whether those situations ever happened to them on a binary scale (0 - No, 1 - Yes). The total score is calculated by averaging all items.

iNAR-5-ESM is an instrument for ESM of intentional non-adherence created for this study. In our previous study^3^, we administered a broader list of 22 non-adherence behaviors with participants responding to a more nuanced scale (1- It has never happened to me, 2 - It has happened to me more than a year ago, 3 - It has happened to me in the past year, 4 - It has happened to me during past two weeks). To best approximate the frequency of iNAR behaviors in a two-week timeframe, we binarized all items such that the value 1 was assigned to responses “Happened during past two weeks” and the value 0 to all other responses. Since many items were highly infrequent (mean below 5%), we decided to either merge items (for items that referred to similar types of behavior) or remove them from the instrument entirely (in case they could not be merged with any other items). After checking the factor structure of the questionnaire and removing one more item, we ended up with five items that converged toward a single common factor. For a full description of the item selection process, see Supplement 1 at https://osf.io/8utgm. In an ESM setting, participants respond to items by stating whether they exhibited the described behaviors that day or not (0 - No, 1 - Yes). The total score is calculated by averaging all items.

TCAM-22^10^ is a self-report instrument assessing the lifetime use of traditional, complementary, and alternative medicine practices. Responses are given in a binary format (0 - *I have never used this practice,* 1 - *I have used this practice* or *I am still using it*), and a total score is calculated by averaging all items. Apart from the total score, four domain scores referring to *alternative medical systems* (7 items), *new age medicine* (5 items), *natural-product-based practices* (5 items), and *rituals/customs* (5 items) can also be calculated by averaging corresponding items.

TCAM-9-ESM is an ESM instrument assessing traditional, complementary, and alternative medicine use created for this study based on our previous data^10^. Using an initial list of 71 TCAM practices for which respondents indicated whether they have used them and when (options: never heard about it/never used it/more than a year ago/in the past year/during the past two weeks), we binarized items to reflect TCAM use within two weeks (1 - *Used in the past two weeks*, 0 - *all other responses*). A vast majority of items were highly infrequent (below 5%), so we opted to either entirely remove them or, when meaningful, merge several items referring to similar practices. After additionally removing two items that did not load on the common factor, we came up with a 9-item solution. The whole procedure of item selection is described in detail in Supplement 2 at https://osf.io/6fmuh. In an ESM setting, participants respond to items by stating whether they used a given TCAM practice that day or not (0 - No, 1 - Yes), and the total score is obtained by averaging all items.

#### Socio-demographic and health-related variables

We collected data on age, gender, education, urban/rural place of residence, household size, number of children under 18 living with the respondent, marital status, and monthly income. To assess the presence of chronic illness, we used a checklist of chronic conditions (e.g. cardiovascular, gastrointestinal). The respondents could select all that apply to them. For those who did not wish to share this information (N = 6), we performed regression imputation for the missing values and rounded decimals to the nearest whole number. We also collected data on smoking, i.e. the number of cigarettes a respondent smoked the previous day. BMI was calculated post hoc based on self-reported weight and height.

#### Personality traits and thinking dispositions - distal predictors

We assessed basic personality structure using the HEXACO-60 inventory^52^ (Serbian version in^53^). Each of the six traits (Honesty/Humility, Emotionality, eXtraversion, Agreeableness, Conscientiousness, and Openness) was captured with ten items, measured on a 5-point scale (1 - *completely agree*; 5 - *completely disagree*).

Disintegration was assessed using a 20-item version of the DELTA9 scale ^16^. The participants indicate their agreement on a 5-point scale (1 - *completely disagree*; 5 - *completely agree*).

Thinking styles were assessed with a short, 8-item version^54^ of the Rational-Experiential Inventory-40^55^. The scale consists of two subscales: rational (e.g. I have a logical mind.) and experiential (e.g. I like to rely on my intuitive impressions.), with four items each. The participants indicate their agreement with the items on a 1 (*definitely not true of myself*) to 5 (*definitely true of myself*) scale.

Cognitive reflection was assessed using the short cognitive reflection test (CRT^56^), consisting of three items that cue an intuitive but incorrect response. A total score was calculated as the proportion of correct responses. After responding to each of the CRT items, participants were asked to express their degree of confidence in that answer on an 11-point rating scale, ranging from 0 to 100% in steps of 10%. A confidence score is expressed as the mean percentage of confidence judgment across the three items.

#### Irrational mindset - proximal predictors

Apophenia was measured using the Snowy Pictures Task consisting of 24 stimuli, each containing a grainy image^57^. Half of the stimuli contain an embedded object within the grainy image and are used as a distractor, while the other half do not. Participants were exposed to stimuli in random order. For each stimulus, participants were instructed to indicate if they see any object in the image and, if so, which object it is. The score is computed as a sum of patterns observed in the images without an embedded pattern (0–12).

Belief in conspiracy theories was measured using two scales. The Conspiracy Mentality Questionnaire (CMQ^58^; Serbian version in^59^), which consists of five items, was used as a measure of a general propensity towards conspiracist ideation (e.g. I think that government agencies closely monitor all citizens). We also included a more specific measure of belief in medical conspiracy theories (adapted from^23, 59^), consisting of five items (e.g. The cure for cancer has been known for some time, but the pharmaceutical industry is hiding it from the public). Participants answered using a 5-point scale on both measures (1 - *completely disagree*; 5 - *completely agree*).

We assessed magical health beliefs with 10 items (e.g. An imbalance between energy currents lies behind many illnesses.) from the general magical beliefs factor of the Magical Beliefs about Food and Health Scale^60^. The participants indicated their agreement on a 5-point scale (1 - *completely disagree*; 5 - *completely agree*).

To measure superstition, we used five items from the Superstition scale^61^ with the highest loadings on the general factor (e.g. I never walk underneath a ladder, even if it means I need to walk a longer distance). The participants indicated their agreement on a 5-point scale (1 - *completely disagree*; 5 - *completely agree*).

Doublethink was measured via the Proneness to doublethink scale^62^. The scale consists of 11 pairs (22 items in total) of contradictory beliefs, and participants indicated their agreement with each of the items on a 4-point scale (1 *- completely disagree;* 4 - *completely agree*). The score was calculated by counting the number of contradictory pairs where participants indicated both statements in the pair to be true (mark 3 or 4 on a 4-point scale). We additionally included ten buffer items that were not scored (five in each of the two blocks that the scale is presented in).

Personal irrational beliefs were assessed via the short General Attitude and Belief Scale (GABS^63^). The scale consists of six items (e.g. It’s awful to have hassles in one’s life and it is a catastrophe to be hassled), measured on a 5-point scale (1 - *completely disagree*; 5 - *completely agree*).

Overconfidence bias was calculated as the difference between the mean confidence score, which ranged between 0 and 100, and the percentage of correct answers on the cognitive reflection test^56^, with a theoretical range of − 100 to 100.

The illusory correlation was assessed by two items. In the first, participants were presented with a 2 × 2 contingency table showing no correlation between the appearance of knee pain and weather changes^64^. In the second, a frequency distribution indicating no correlation between drug admission and healing was presented in textual form. Responses indicating a positive correlation between variables were treated as indicating the presence of an illusory correlation. The score was calculated as a proportion of biased responses, ranging from 0 to 1.

Naturalness bias was measured as a hypothetical preference for natural over synthetically generated mineral water, all other things being equal^65^; the score ranged from 0 to 1.

Belief bias was measured using four syllogistic reasoning problems that conflicted with the empirical and the logical status of the conclusion. The measure of bias was calculated as a proportion of answers indicating that participants based their judgments about the conclusion’s validity on the conclusion’s believability^66^, thus ranging from 0 to 1.

Commitment bias was measured as a preference to continue advocating the health benefits of using lard rather than sunflower oil in the diet despite the new evidence showing no difference between them; the score ranged from 0 to 1.

Probabilistic reasoning biases were assessed by two items indicating neglect of base rates^28^, one item indicating gambler’s fallacy, and one indicating misperception of the chance^67^. The score was a proportion of biased answers ranging from 0 to 1.

#### Socio-political beliefs - proximal predictors

To measure (mis)trust in the healthcare system and professionals, we selected items from the Women’s Trust and Confidence in the Healthcare System - WITCH scale^68^. We used two items with the highest loadings from the Interpersonal trust dimension to assess trust in health professionals, and two items with the highest loadings from the Generalized mistrust dimension to assess mistrust in the healthcare system. Participants expressed their agreement on a 5-point scale (1 - *completely disagree*; 5 - *completely agree*).

Trust in science was assessed with two items (e.g. The scientific method is the best way to reach the truth.), to which participants responded using a 5-point scale (1 - *completely disagree*; 5 - *completely agree).*

We assessed religiosity and spirituality with a single item each (I consider myself to be a religious/spiritual person), on a 5-point scale (1 - *completely disagree*; 5 - *completely agree).*

Political orientation was assessed with a single item (1 - *far left*; 4 - *center*; 7 - *far right*).

The majority of measured variables had satisfactory levels of reliability (*α*s > 0.70, see Supplementary Table S2 at https://osf.io/967hk?view_only=None), except for mistrust in the healthcare system (*α* = 0.58), GABS (*α* = 0.65), and, expectedly, due to their shortness, several measures of cognitive biases (*α*s < 0.60; see, e.g. Ref.^28^).

### Analytical strategy

We report descriptives and reliabilities of all instruments. To explore the relations between retrospectively assessed variables we used the Pearson correlation coefficient, while for exploring the relations between daily and retrospective health behaviors assessment and their relations to psychological roots, we employed a multilevel approach. Unlike ordinary least squares (OLS) models, the multilevel approach is specifically designed for hierarchically structured data. As days of assessment (level 1) are nested within individuals (level 2), multilevel random coefficient modeling (MRCM) was the most appropriate method for our data^69^. The main advantage of MRCM is the possibility to model random effects, which means calculating more accurate parameter estimates and tests of significance compared to OLS^70^. It also enables the decomposition of the overall variance into variance from stable factors (individual differences) and variance from unstable factors (intra-individual differences).

We tested several multilevel random coefficient models, with iNAR and TCAM measured at level-1 (repeatedly for 14 days within a random effects model) as dependent variables. To better understand the sources of variations in daily TCAM use and iNAR engagement (i.e. the amount of intra- and interindividual variation), we first tested the basic unconditional model (i.e. a model with no predictors which only includes the intercept) for both dependent variables (Model 1). Second, since Pearson correlations would be meaningless for hierarchically structured data, we investigated the relation between TCAM and iNAR by predicting daily iNAR engagement based on daily TCAM use (Model 2). To determine the concordance of two different assessment methods, in Model 3 we used retrospective reports of TCAM/iNAR as predictors of corresponding ESM variables.

To discover the best predictors of questionable health behaviors, we proceeded to test three additional models (Models 4–6), for each of the three large predictor groups separately. In Model 4 we started with the historically most investigated set of TCAM/iNAR predictors - sociodemographic and health-related variables. In Model 5, we explored the relationships between distal psychological variables (personality traits and thinking dispositions) and TCAM/iNAR, whilst, in Model 6 we focused on the relationships between proximal psychological variables (irrational mindset and socio-political variables) and TCAM/iNAR. In addition, to compare the precursors of daily to those of lifetime variations in questionable health behaviors, we tested the same three sets of variables (Models 4–6) as predictors of retrospectively assessed TCAM/iNAR as dependents, using multiple regression analyses. Lastly, as per reviewer’s recommendation, we tested the bivariate relationships between distal and proximal predictors and dependent variables using multi-level random coefficient modeling. We used HLM 6.06 software^71^ for MRCM and SPSS v.23 for all other analyses. Scripts with equations for tested models are accessible at https://osf.io/f95up?view_only=c11adfeea7af4d93af0e147399aaeafd.

### Deviations from the preregistration

Here we list all deviations from the pre-registration. First, we have reordered the pre-registered hypotheses, so they follow the flow of the paper better. Second, instead of using only three items with the highest loadings on the first and second factors, we assessed magical health beliefs with all 10 items from the general magical beliefs factor of the *Magical Beliefs about Food and Health Scale*^60^. We also extended the list of sociodemographic variables to include: urban/rural place of residence, household size, marital status, and the number of children under the age of 18. Finally, we averaged the scores on three cognitive biases (base rate neglect and gambler’s fallacy, and misperception of chance, i.e., hot hand fallacy) for a new aggregate measure of the *Probabilistic reasoning bias*.

## Results

We observed high rates of questionable health practices during the period of 14 days. Among TCAM practices, the most frequent was the consumption of herbal products and consumption of supplements, with 75% of participants reporting at least one use over a 14-day period (see Table 1) which corresponded to over 80% of respondents retrospectively reporting at least one usage in the course of a lifetime (see Supplementary Table S1 at https://osf.io/967hk?view_only=None). These two practices had a bimodal distribution, with participants either using them every day over 14 days or not using them at all. This is also reflected in their higher dispersion (see the last column of Table 1). Other TCAM practices were less frequent and positively skewed.

**Table 1:** Percentage of behavior or practice use over a 14-day period (overall use; *N*observations = 3136) and percentage of participants that indicated at least one use of given behavior or practice (prevalence; *N* = 224) over a 14-day period.

Level 1 items	Overall use	Prevalence	SD
TCAM-ESM			
Use of alternative treatments or following advice from an alternative practitioner	5.1	27.2	12.8
Use of mind-body exercises	8.3	32.1	16.7
Use of religious healing	22.6	52.7	32.3
Use of art therapies	14.7	36.2	26.6
Use of meditation or other relaxation practices	9.9	34.4	20.1
Use of herbal balms or similar products	28.0	58.5	33.6
Consumption of herbal-based products like teas	50.4	76.3	39.4
Use of specific eating regimes	13.9	31.7	28.1
Use of supplements like vitamins without a recommendation from a physician	45.9	75.4	38.8
iNAR-ESM			
Skipping a medical checkup	4.9	20.1	16.1
Ignoring symptoms	10.9	44.2	18.1
Taking non-prescribed drugs	6.3	25.4	15.5
Adjusting/changing medication on own accord	2.3	12.1	8.7
Not taking prescribed therapy	5.0	24.6	12.4

*TCAM* traditional, complementary, and alternative medicine, *iNAR* intentional nonadherence to official medical recommendations. *SD* is calculated after aggregating data for participants.

The lowest, but still not negligible TCAM prevalence was observed for the use of alternative treatments (27% of respondents indicated at least one use in 14 days), specific eating regimes (28%), and mind-body exercises (32%), which roughly corresponded to their reported use in retrospective reports.

The most frequent iNAR behavior was ignoring symptoms that required a visit to a doctor (44% of respondents reported doing that at least once in a 14-day period), whilst the least frequent was adjusting/changing medication on one’s own accord (reported at least once by 12% respondents). Taken together, iNAR behaviors were in general less frequently reported in daily assessments than retrospectively.

Intercorrelations among all measured variables and retrospectively assessed TCAM and iNAR are provided in Supplementary Table S3 at https://osf.io/967hk?view_only=None. Results showed that the two categories of questionable health behaviors were weakly, but positively correlated, as observed in previous research^10, 72^.

As detailed in the Analytical strategy, we tested six multilevel models with daily TCAM/iNAR behaviors as dependents, as well as additional OLS regression models with retrospectively assessed TCAM/iNAR behaviors as dependent variables for models 4-6. We consider all our predictions (H1-H8) for the daily and retrospective TCAM/iNAR behaviors in parallel.

Model 1 (unconditional) showed that approximately half of the iNAR variance could be ascribed to intraindividual differences, i.e. daily variations, and the other half to the differences between individuals (Table 2). In the case of TCAM, intraindividual variations explained 30% of the overall variance, whilst 70% of the variance could be ascribed to the differences between individuals. Model 2 tested the relationship between daily engagements in the two categories of questionable health practices. Similarly to what we observed in the retrospective assessment of these practices (*r* = 0.30, *p* < 0.001), the relationship was positive albeit smaller in magnitude (TCAM predicted iNAR with *b* = 0.09, *p* = 0.002). Model 3 demonstrated that daily engagement in questionable health practices was successfully predicted by their lifetime use; this relationship was stronger for TCAM (*b* = 0.61, *p* < 0.001) compared to iNAR behaviors (*b* = 0.10, *p* = 0.001). This can be due to (a) the larger discrepancy in behavior frequency tracked daily and retrospectively for iNAR (6% vs. 37% for daily and lifetime engagement, respectively) than for TCAM (22% vs. 36%), (b) the difference in the percentage of the variance of daily engagement which can be attributed to interpersonal variations (47% for iNAR and 70% for TCAM), but also (c) to the fact that the use of TCAM includes preventive use, providing more opportunities to engage in this type of behavior than iNAR.

**Table 2:** Relationship between daily and retrospectively assessed iNAR and TCAM.

	Multi-level random coefficient modeling			
	iNAR_ESM		TCAM_ESM	
ICC (model 1 - unconditional model)	0.47		0.70	
Level 1 (model 2)	B	t_(223)_	B	t_(223)_
iNAR-ESM	–	–	–	–
TCAM-ESM	0.09**	3.16	–	–
Level 2 (model 3)	B	t_(222)_	B	t_(222)_
iNAR (retrospective)	0.10**	3.47	–	–
TCAM (retrospective)	–	–	0.61**	7.11

*iNAR* intentional non-adherence to medical recommendations, 12-item scale; *TCAM* traditional complementary alternative medicine, 22-item scale; *iNAR*-*ESM* intentional non-adherence to medical recommendations, 5-item scale administered daily, during a 14-day period; *TCAM*-*ESM* traditional complementary alternative medicine, 9-item scale, administered daily, during a 14-day period; *ICC* intraclass correlation coefficient; % - percent of explained variance.

**p <0.01.

Model 4 showed that sociodemographic variables are of little importance for both types of questionable health practices assessed daily (Table 3). Whilst we expected gender to be predictive of TCAM (H1), the only significant predictor here was the number of chronic illnesses, and that was only for iNAR.

**Table 3:** Predicting iNAR and TCAM - assessed daily and retrospectively - by sociodemographic variables (multivariable analysis).

	Multi-level random coefficient modeling				Multiple regressions			
	iNAR_ESM		TCAM_ESM		iNAR (retrospective)		TCAM (retrospective)	
Socio-demographics (model 4)	B	t_(213)_	B	t_(213)_	β	t_(213)_	β	t_(213)_
Sex	– 0.00	− 0.23	0.04	1.57	0.00	0.03	0.19***	2.64
Age	− 0.00	− 0.054	0.00	− 0.42	− 0.09	− 0.12	− 0.05	− 0.71
Urban/rural	− 0.01	− 0.20	− 0.01	− 0.46	0.05	0.78	0.07	1.07
Education	− 0.01	− 1.84	0.00	0.14	− 0.14	− 0.1.89	0.16*	2.34
Marital status	− 0.01	− 0.07	− 0.01	0.63	− 0.06	− 0.079	0.03	0.42
Income	− 0.00	− 0.80	0.00	− 1.07	− 0.03	− 0.37	− 0.01	− 0.18
Household size	0.00	0.20	0.01	− 0.91	0.03	0.34	0.03	0.38
Children under 18 years	− 0.01	− 0.80	0.02	1.14	− 0.03	− 0.42	0.03	0.37
BMI	0.00	0.81	0.00	0.06	0.14	1.81	0.00	0.06
Number of chronic conditions	0.02**	3.08	0.02	1.91	0.11	1.56	0.10	1.49
Percentage of explained variance	3.2%		0.4%		1.0%		4.5%	

*iNAR* Intentional non-adherence to medical recommendations, 12-item scale; *TCAM* traditional complementary alternative medicine, 22-item scale; *iNAR*-EiSM intentional non-adherence to medical recommendations, 5-item scale administered daily, during a 14-day period; *TCAM*-*ESM* traditional complementary alternative medicine, 9-item scale, administered daily, during a 14-day period; *ICC* intraclass correlation coefficient; % Percent of explained variance.

^*^p <0.05, **p <0.01, ***p <0.001.

Model 5 demonstrated the importance of distal psychological variables - basic thinking styles and personality traits (Table 4). Cognitively less reflective and those more prone to psychotic-like experiences and behaviors (higher on disintegration trait) and impulsivity (lower on Conscientiousness trait), were more likely to engage in daily iNAR. Those higher on disintegration (H2a) and less cognitively reflective (H3b), more extroverted and agreeable were also more prone to daily TCAM practices. When all other variables were accounted for, we found no significant contribution of either experiential (H2b) or analytical thinking style (H3a). When looking at these relationships separately, though, more experiential individuals did tend to use TCAM more often - this was in line with H2b (Supplementary Table S4 at https://osf.io/967hk?view_only=None).

**Table 4:** Predicting iNAR and TCAM - assessed daily and retrospectively - by distal psychological variables (multivariable analysis).

	Multi-level random coefficient modeling				Multiple regressions			
	iNAR_ESM		TCAM_ESM		iNAR (retrospective)		TCAM (retrospective)	
Distal psychological predictors (model 5)	B	t_(213)_	B	t_(213)_	β	t_(213)_	β	t_(213)_
Honesty	0.01	0.86	−0.01	−0.81	−0.24***	−3.52	−0.17*	−2.33
Emotionality	0.01	1.10	0.02	1.04	−0.03	−0.48	0.11	1.64
Extraversion	0.01	0.46	0.05**	2.77	−0.11	−1.61	0.18**	2.64
Agreeableness	−0.00	−0.24	0.04*	2.42	−0.02	−0.37	0.11	1.71
Conscientiousness	−0.02*	−2.00	−0.01	−0.26	−0.20**	−2.88	0.07	0.95
Openness	0.01	0.66	0.02	1.05	0.13	1.92	0.09	1.27
Disintegration	0.03*	2.34	0.04*	2.25	0.17*	2.21	0.24**	3.07
REI-Rationality	−0.00	−0.48	−0.01	−0.95	−0.02	−0.24	0.05	0.67
REI-Experientiality	−0.01	−0.93	0.02	1.56	0.10	1.48	0.08	1.17
CRT	−0.04**	−2.64	−0.10**	−3.62	0.02	0.34	−0.20**	−3.01
Percentage of explained variance	7.8%		15.6%		24.1%		17.4%	

*iNAR* intentional non-adherence to medical recommendations, 12-item scale; *TCAM* traditional complementary alternative medicine, 22-item scale; *iNAR*-*ESM* intentional non-adherence to medical recommendations, 5-item scale administered daily, during a 14-day period; *TCAM*-*ESM* traditional complementary alternative medicine, 9-item scale, administered daily, during a 14-day period; *ICC* intraclass correlation coefficient; % Percent of explained variance.

^*^p <0.05, **p <0.01, ***p <0.001.

Finally, Model 6 demonstrated the importance of proximal psychological variables for daily TCAM and iNAR behaviors (Table 5). Expectedly, this set of variables - containing various measures of irrational thinking and beliefs - was of greater predictive value for TCAM compared to iNAR (H6). Contrary to our predictions, however, when iNAR was assessed daily, none of the proximal variables predicted it significantly, while as expected, daily TCAM behaviors were predicted by magical health beliefs (H4b). Judging by the t-statistic (since the regression weights were unstandardized), it was also the best predictor (in line with H5).

**Table 5:** Predicting iNAR and TCAM - assessed daily and retrospectively - by proximal psychological variables (multivariable analysis).

	Multi-level random coefficient modeling				Multiple regressions			
	iNAR_ESM		TCAM_ESM		iNAR (retrospective)		TCAM (retrospective)	
Proximal psychological predictors (model 6)	B	t_(213)_	B	t_(213)_	β	t_(213)_	β	t_(213)_
Religiosity	− 0.00	− 0.44	0.02*	2.63	− 0.16*	− 2.18	0.20**	2.85
Spirituality	0.00	0.72	0.00	0.41	0.05	0.71	0.07	1.13
Trust in health professionals	− 0.01	− 1.20	− 0.01	− 0.60	− 0.21*	− 2.35	− 0.02	− 0.19
Mistrust in the healthcare system	− 0.01	− 1.23	0.00	0.25	0.10	1.34	0.09	1.27
Trust in science	0.00	0.04	0.02	0.86	0.02	0.27	0.03	0.37
Political orientation	0.00	0.14	− 0.01	− 0.93	0.06	0.91	− 0.04	− 0.57
GABS	− 0.00	− 0.37	0.00	− 0.13	0.15*	2.14	0.10	1.49
Superstition	0.01	1.57	0.02*	2.00	0.20**	2.74	0.19**	2.66
Conspiracy	− 0.02	− 1.41	− 0.03	− 1.35	0.02	0.21	− 0.05	− 0.52
Medical conspiracy	0.02	1.70	0.00	0.03	0.03	0.24	0.03	0.29
Magical health beliefs	0.01	0.71	0.06**	3.02	0.19*	2.23	0.21^*^	2.48
Doublethink	− 0.00	− 0.78	− 0.01*	− 2.27	− 0.07	− 0.95	− 0.09	− 1.35
Overconfidence on CRT	0.00	1.15	0.00	1.17	− 0.18*	− 2.52	0.10	1.45
Naturalness bias	− 0.00	− 0.26	0.04	1.25	0.02	0.28	0.00	− 0.07
Commitment bias	− 0.00	− 0.30	0.01	0.59	0.00	− 0.02	0.02	0.27
Illusory correlations	0.03	1.49	0.01	0.17	− 0.01	− 0.11	− 0.03	− 0.46
Belief bias	0.00	0.13	0.10*	2.57	− 0.05	− 0.76	0.00	0.04
Probability bias	0.01	0.41	− 0.02	− 0.39	0.02	0.24	0.05	0.82
Apophenia	0.00	0.52	0.01	1.70	0.11	1.64	0.08	1.27
Percentage of explained variance	4.8%		22.5%		19.9%		24.8%	

*iNAR* intentional non-adherence to medical recommendations, 12-item scale; *TCAM* Traditional complementary alternative medicine, 22-item scale; *iNAR*-*ESM* intentional non-adherence to medical recommendations, 5-item scale administered daily, during a 14-day period; *TCAM*-*ESM* traditional complementary alternative medicine, 9-item scale, administered daily, during 14-days period; ICC intraclass correlation coefficient;

% Percent of explained variance.

*p <0.05, **p <0.01.

We did not find significant relations to either higher belief in medical conspiracy theories (H4a) or proneness to naturalness bias (H4c) when accounting for other predictors, however, they did predict TCAM in the expected direction in bivariate analyses (Supplementary Table S5 at https://osf.io/967hk?view_only=None). Daily TCAM was also predicted by higher superstition and, as we expected, religiosity (H8) and more proneness to belief bias. Surprisingly, it was also predicted by lower proneness to doublethink, likely due to a suppressor effect - doublethink showed a positive zero-order correlation with TCAM behaviors (*r* = 0.16). Overall, the pattern of results observed when we tested the relationship between distal and proximal predictors and daily iNAR and TCAM use through bivariate MRCM analyses was very similar to the results of multivariate analyses (see Supplementary Tables S4 and S5 on https://osf.io/967hk?view_only=None).

When looking at TCAM and iNAR assessed retrospectively, our results show that, in line with our predictions (H1), women were more likely to use TCAM. Education was also a significant predictor of retrospective TCAM use, while none of the sociodemographic variables contributed to the prediction of retrospective iNAR. As for the distal predictors, lower conscientiousness and honesty predicted retrospective iNAR, while higher disintegration (in line with H2a) and lower cognitive reflection (in line with H3b) were related to more retrospective TCAM use. Retrospective TCAM use was also independently predicted by higher extraversion and lower honesty. Similar to daily TCAM use, we found no significant contribution of either rational or experiential thinking style (H2b, H3a). As opposed to daily tracked iNAR behaviors, proximal predictors tended to explain a substantial amount of variance in retrospectively assessed iNAR (19.9%), with higher magical health beliefs, superstition, personal irrational beliefs, religiosity, and overconfidence contributing to the prediction. As expected (H7), trust in the healthcare professionals was one of its main predictors, however, we found no contribution of mistrust in the healthcare system. In the case of retrospective TCAM, in line with our hypothesis (H4b) magical health beliefs contributed significantly to its prediction, alongside superstition, though medical conspiracies (H4a) and naturalness bias (H4c) did not. We also found that more religious individuals were also more likely to use TCAM (H8).

To summarize, the relevance of gender for TCAM (H1) was partially supported by our data: women were more prone to retrospectively, but not to daily assessed TCAM practices. We only treated hypotheses on the relationships between variables as supported by the data when there was a unique contribution of a predictor to a criterion, i.e. when the beta weight of a predictor in the regression function was significant. Next, we found partial support for H2 and H3 concerning cognitive reflection and disintegration, but not regarding rational/experiential thinking styles: higher disintegration and lower cognitive reflection contributed to TCAM behaviors, both daily and retrospectively assessed. As expected, magical beliefs about health were the best predictor of TCAM, regardless of the assessment method (H5). As for other irrational beliefs whose contribution we expected (H4), although their zero-order correlations with TCAM were in the expected direction, neither endorsement of medical conspiracy theories nor naturalness bias had a unique contribution to TCAM use.

Irrational mindset variables were more important for TCAM than iNAR, especially for daily assessed behaviors, in line with H6. As for H7, it was partially supported - only mistrust in health professionals played a role in iNAR, and only when iNAR was retrospectively assessed. Furthermore, we observed the expected relation between both daily and retrospective TCAM use and religiousness - it was more frequent in religious people (H8).

## Discussion

Our results clearly show that both questionable health behaviors - iNAR and TCAM - are quite frequent: almost all respondents (99%) reported having used TCAM at least once in a lifetime or having at least once deliberately disregarded medical recommendations (94%). This is in line with the prevalence of these behaviors observed in previous research^3, 7, 10^. In this study, we further explored the frequency of both health behaviors within a two-week time window by their daily assessment.

Natural products-based practices were the most frequent TCAM practices, followed by religious rituals, and finally, practices that are typically classified as belonging to the new age and alternative medical systems^5, 10^.

The observed difference between the two assessment methods was larger in the case of iNAR (average use of the listed practices - 6% daily vs. 37% retrospectively) than TCAM (average use of the listed practices - 22% daily vs. 36% retrospectively). These findings likely reflect the fact that TCAM is predominantly used preventively^10^ and that it encompasses a larger set of practices, thus providing more opportunities to engage in this type of behavior in the short term. On the other hand, iNAR mostly requires the presence of current health-related problems and a consequent decision to disregard medical recommendations. Intraindividual, daily variations in iNAR behaviors were larger (around 50%) than variations in TCAM behavior (around 30%). For TCAM, scores obtained by retrospective assessment were better predictors of daily engagement than was the case for iNAR scores. Taken together, our results show that, compared to iNAR, daily TCAM practices appear to be more frequent, slightly more consistent, and more unequivocal across the methods of assessment, thus reflecting individual differences to a larger extent.

A weak but stable tendency that those prone to TCAM also engage in iNAR more frequently (e.g. Ref.^10, 72^) was replicated here across both methods of assessment. This further supports the idea that questionable health behaviors are mutually related, i.e. that people prone to TCAM use are also more prone to intentionally ignore official medical recommendations.

Sociodemographic variables were of far less importance for both questionable health practices, compared to psychological variables: only gender (female) proved to be predictive, and still only of TCAM. Regarding distal psychological predictors, lower cognitive reflection and higher disintegration appeared to be the most robust predictors of both questionable health behaviors, independent of the method of assessment.

Variables reflecting irrational mindset (proneness to conspiracism, magical health beliefs, and religiousness) were repeatedly the most important correlates of TCAM, independent of the assessment method. In the case of iNAR, the signature of proximal predictors was recognizable only when it was assessed retrospectively - elements of irrational mindset (proneness to conspiracism, magical health beliefs, and personal irrational beliefs), cognitive underconfidence, less trust in health care professionals and lower religiousness appeared to be relevant; these predictors did not emerge for daily iNAR. We attribute this to the fact that iNAR behaviors showed more daily (intraindividual) variations than TCAM behaviors, meaning that TCAM behaviors are more stable within the individual, which makes them more likely to be the effect of stable dispositions such as irrational mindset. Additionally, a relatively low prevalence of almost all daily assessed iNAR behaviors in our sample restricted their variability, thus decreasing the potential explanatory power of distal and proximal predictors.

Finally, proximal psychological variables (irrational mindset and socio-political attitudes) appeared to be more relevant in predicting TCAM than the distal ones (basic personality traits, thinking styles, and dispositions), whilst the opposite was true for iNAR (distal predictors were more relevant). This is in line with our previous findings^3, 10^. Taken together, these results highlight the importance of the irrational mindset in TCAM and its less importance for iNAR.

### Implications

Our study demonstrates that daily variations in both types of questionable health practices can be reliably assessed and leaves future researchers with two viable novel instruments for such assessment. We observed robust behavioral tendencies and were able to consistently relate them to specific predictors across different methods. A prospective, within-person approach we applied, contrasting the usual between-person approach^41^, comes to the fore when studying health-related behaviors. Tracking the health behaviors of individuals allows for monitoring the effectiveness of interventions at the within-person level. In addition, while typical non-adherence studies and the majority of TCAM studies looked into specific behaviors in clinical populations, this study measured a more general behavioral pattern that could be observed in the general population with non-trivial prevalence. The results could thus translate to a more universal input for patient-physician communication: knowing the psychological profile of people prone to these widespread types of non-adherence and widespread types of TCAM use could help physicians tailor the messages to broader groups of patients, i.e. gives the physicians a more feasible task in comparison to tailoring it to clinical subgroups.

### Limitations and future research

Although ESM enables higher representativeness of the results due to its ecological validity, and we strategically recruited participants from the general population, the generalizability of our results is limited to some extent. It would be informative to replicate the observed patterns in clinical samples consisting of individuals who engage in health practices more often, i.e. who have more opportunity to not adhere to official recommendations or use TCAM (e.g. cancer patients, chronic pain patients, diabetics).

In this study, we opted for a fixed daily prompt for ambulatory assessment, whilst future studies could use event-based triggering. We used a relatively narrow list of TCAM and iNAR practices, and these could be extended. It is, however, always a trade-off in the experience sampling assessment because the participants must repeatedly respond to the same questionnaire, thus one needs to make sure they stay motivated. Our assessment of health status was also quite straightforward, and it could be measured more extensively in future studies. Similarly, a number of brief scales we employed (e.g. mistrust in the healthcare system) showed low internal reliability thus limiting our insights on their predictive power - their longer versions might prove to be more reliable and, consequently, related to TCAM and iNAR to a higher degree.

## Conclusion

To the best of our knowledge, this is the first multi-method study to simultaneously assess two broad categories of questionable health behaviors in the general population: we tracked intentional non-adherence to medical recommendations and use of TCAM via experience sampling and via traditional, retrospective assessment. Across both methods of assessment, two categories of questionable health behaviors were weekly but positively related: people prone to iNAR were simultaneously prone to TCAM practices. This is also the first study to relate these practices to a broad set of psychological predictors, encompassing basic personality traits and thinking styles, but also to an irrational mindset - a set of logically non-normative beliefs and biases. Although psychological predictors were far more relevant than socio-demographic for both targeted behavioral categories, their psychological signature was different: while iNAR was mostly rooted in basic personality traits, an irrational mindset played a substantial role in the use of TCAM practices.

### Supplementary Information

Supplementary Tables.

## Data Availability

Data used in the study are available on the Open Science Framework project page (https://osf.io/4dq9m/?view_only=c11adfeea7af4d93af0e147399aaeafd).
