# Supplementary material for: Tracking variations in daily questionable health behaviors and their psychological roots: a preregistered experience sampling study
Source: Sci Rep. 2023 Aug 28;13:14058. doi: 10.1038/s41598-023-41243-w (PMC10462719; doi:10.1038/s41598-023-41243-w)
Supplement: Supplementary file 1 — Supplementary Tables. [file 41598_2023_41243_MOESM1_ESM.docx]

**Supplementary materials**

Table S1. *Percentage of participants reporting lifetime use of a given behavior or practice (Level 2; N = 224)*

| **Level 2 items** | % | SD |
| --- | --- | --- |
| **TCAM** |  |  |
| Acupuncture | 10.3 | 30.4 |
| Homeopathy | 18.3 | 38.8 |
| Quantum medicine or related techniques | 6.3 | 24.3 |
| (Bio)energetic therapies | 16.1 | 36.8 |
| Crystal therapy (including wearing healing crystals) | 5.8 | 23.4 |
| Chiropractic, osteopathy, bonesetters | 25.4 | 43.7 |
| Special types of massages (including shiatsu, hot stones massage, marma point massage, meridian massage therapy, tuina, rolfing massage, hydrotherapy, Kneipp therapy, Trigger-point massage, lymphatic drainage massage, etc.) | 24.1 | 42.9 |
| Mind-body exercises (e.g., yoga, qigong, taichi, pilates) | 27.2 | 44.6 |
| Spiritual healing or rituals | 3.1 | 17.4 |
| Art therapy, musical therapy, voice therapy, sound therapy, or dance therapy | 14.7 | 35.5 |
| Meditation, mindfulness, progressive relaxation, other relaxation techniques, and breathing exercises | 22.3 | 41.7 |
| Guided fantasy/imagination or visualization | 8.0 | 27.2 |
| Products of herbal origin (e.g., teas, drinks, extracts, drops, essences, tinctures made of plants such as comfrey, chamomile, ginger, garlic, aloe vera, etc.) | 93.3 | 25.1 |
| Herbal balms, pledgets, creams, or ointments (e.g., garlic, marigold, lavender, yarrow, olive oil) | 87.5 | 33.1 |
| Consumption of herbs (e.g.,  garlic, house leek, dried figs, cornel) or honeybee products (honey, honeybee pollen, honeycomb, propolis) | 87.5 | 33.1 |
| Vitamins, minerals, or antioxidants (without a recommendation from a physician) | 84.4 | 36.4 |
| Supplements, probiotics, or prebiotics (without a recommendation from a physician) | 83.0 | 37.6 |
| Wearing pendants, amulets, or talismans | 19.2 | 39.5 |
| Wearing red thread or string around the hand | 20.1 | 40.2 |
| Lead pouring (fear pouring) | 19.6 | 39.8 |
| Water from mineral (healing) springs or holy water | 64.3 | 48.0 |
| Praying for own health, visiting churches or monasteries | 58.0 | 49.5 |
| **iNAR** |  |  |
| Felt symptoms for which people usually go to the doctor, but waited for these symptoms to pass instead of going to the doctor | 83.5 | 37.2 |
| Did not report all of my symptoms or minimized them when reporting to a doctor | 27.2 | 44.6 |
| Took antibiotics even though a doctor did not prescribe them tome | 57.1 | 49.6 |
| Took an anxiolytic even though a doctor did not prescribe it to me | 34.4 | 47.6 |
| Took some other prescription drugs even though a doctor did not prescribe it to me | 40.6 | 49.2 |
| Stopped taking antibiotics earlier than prescribed by a doctor, e.g., when my symptoms were relieved | 31.7 | 46.6 |
| Did not take prescribed therapy. | 29.0 | 45.5 |
| Self-determined the dosage of the prescribed medicine. | 18.8 | 39.1 |
| Decided myself which of the prescribed drugs I will take and which I will not | 24.1 | 42.9 |
| Avoided going to a medical check-up (e.g., a scan) that was recommended to me by a doctor | 17.9 | 38.4 |
| Withdrew from a scheduled medical follow-up | 33.9 | 47.5 |
| Refused to change my lifestyle habits (e.g., my diet or physical activity) as recommended by a doctor | 40.6 | 49.2 |

Note. TCAM – traditional, complementary, and alternative medicine, iNAR – intentional nonadherence to official medical recommendations. SD and range are calculated after aggregating data

Table S2 *Descriptives for all variables*

| **Variables** | *M* | *SD* | No. of items | Reliability |
| --- | --- | --- | --- | --- |
| **Outcomes** |  |  |  |  |
| TCAM-9 ESM (level 1) | 0.22 | 0.17 | 9 | .77 |
| iNAR-5 ESM (level 1) | 0.06 | 0.10 | 5 | .70 |
| TCAM-22 (level 2) | 0.36 | 0.15 | 22 | .75 |
| iNAR-12 (level 2) | 0.37 | 0.23 | 12 | .75 |
| **Distal predictors** |  |  |  |  |
| Honesty | 3.68 | 0.76 | 10 | .79 |
| Emotionality | 3.28 | 0.64 | 10 | .71 |
| eXtraversion | 3.24 | 0.62 | 10 | .72 |
| Agreeableness | 3.18 | 0.68 | 10 | .75 |
| Conscientiousness | 3.85 | 0.64 | 10 | .78 |
| Openness | 3.37 | 0.76 | 10 | .78 |
| Disintegration | 2.24 | 0.68 | 20 | .88 |
| REI R | 3.75 | 0.87 | 4 | .81 |
| REI E | 3.45 | 0.87 | 4 | .77 |
| CRT | 0.38 | 0.44 | 3 | .77 |
| **Proximal predictors** |  |  |  |  |
| Religiosity | 3.39 | 1.38 | 1 | / |
| Spirituality | 2.31 | 1.21 | 1 | / |
| Trust in healthcare professionals | 3.31 | 1.02 | 2 | .82 |
| Mistrust in the healthcare system | 3.56 | 0.96 | 2 | .58 |
| Trust in science | 3.75 | 0.94 | 2 | .76 |
| Political orientation | 4.08 | 1.63 | 1 | / |
| GABS total score | 3.40 | 0.67 | 6 | .65 |
| Superstition | 2.55 | 0.99 | 5 | .72 |
| General conspiracy | 4.02 | 0.75 | 5 | .78 |
| Medical conspiracy | 3.35 | 1.11 | 5 | .87 |
| Magical health beliefs | 3.14 | 0.79 | 10 | .84 |
| Doublethink | 5.84 | 2.65 | 11 | .72 |
| Overconfidence bias | 49.22 | 39.57 | / | / |
| Naturalness bias | 0.81 | 0.39 | 1 | / |
| Commitment bias | 0.46 | 0.50 | 1 | / |
| Illusory correlations | 0.56 | 0.35 | 2 | .13 |
| Belief bias | 0.73 | 0.30 | 4 | .57 |
| Probability reasoning biases | 0.44 | 0.25 | 4 | .45 |
| Snowy pictures | 3.94 | 3.35 | 24 | .84 |

*Note.* Level 1 *N*observations= 3136, Level 2 *N* = 224; TCAM – traditional, complementary, and alternative medicine; iNAR – intentional nonadherence to official medical recommendations; Reliability of the level-1 variables' intercept is calculated as: λ = τ_00_/( τ_00_ + σ/n_k_); τ_00_ – variance of inter-individual differences; σ – variance of intra-individual differences; n_k_ –number of measurement points at level 1. This coefficient gives similar information as the percentage of the variance explained by the inter-individual differences; Reliability of the level-2 variables is Cronbach alpha; * *p <*.05, ** *p <*.01, *** *p <*.001

Table S3 Intercorrelations between all variables in the study (Part 1)

|  |  | 1 | 2 | 3 | 4 | 5 | 6 | 7 | 8 | 9 | 10 | 11 | 12 | 13 | 14 | 15 | 16 | 17 | 18 | 19 | 20 |
| --- | --- | --- | --- | --- | --- | --- | --- | --- | --- | --- | --- | --- | --- | --- | --- | --- | --- | --- | --- | --- | --- |
| DV | 1 TCAM Retrospective |  |  |  |  |  |  |  |  |  |  |  |  |  |  |  |  |  |  |  |  |
|  | 2 iNAR Retrospective | .30** |  |  |  |  |  |  |  |  |  |  |  |  |  |  |  |  |  |  |  |
| Socio-demographics | 3 Gender | .20** | -.04 |  |  |  |  |  |  |  |  |  |  |  |  |  |  |  |  |  |  |
|  | 4 Age | -.04 | -.03 | .03 |  |  |  |  |  |  |  |  |  |  |  |  |  |  |  |  |  |
|  | 5 Urban/Rural | .10 | .05 | -.02 | -.16* |  |  |  |  |  |  |  |  |  |  |  |  |  |  |  |  |
|  | 6 Education | .16* | -.11 | .03 | -.13* | .12 |  |  |  |  |  |  |  |  |  |  |  |  |  |  |  |
|  | 7 Marital status | -.01 | -.02 | -.03 | .21** | -.01 | -.31** |  |  |  |  |  |  |  |  |  |  |  |  |  |  |
|  | 8 Income | -.07 | -.02 | -.12 | .16* | -.11 | -.20** | .19** |  |  |  |  |  |  |  |  |  |  |  |  |  |
|  | 9 Household size | .08 | .01 | .09 | .01 | .07 | -.03 | .23** | .01 |  |  |  |  |  |  |  |  |  |  |  |  |
|  | 10 Children under 18 years | .06 | -.02 | .04 | .02 | .04 | -.12 | .34** | .03 | .54** |  |  |  |  |  |  |  |  |  |  |  |
|  | 11 BMI | -.07 | .14* | -.32** | .25** | -.06 | -.07 | .10 | .00 | -.05 | -.03 |  |  |  |  |  |  |  |  |  |  |
|  | 12 Number of chronic conditions | .09 | .12 | .06 | .30** | -.02 | -.12 | .14* | .08 | .12 | .11 | .19** |  |  |  |  |  |  |  |  |  |
| Distal psychological predictors | 13 Honesty-Humility | -.17* | -.41** | .05 | .13* | -.11 | .03 | .13 | .01 | -.01 | .01 | .10 | .10 |  |  |  |  |  |  |  |  |
|  | 14 Emotionality | .17* | .05 | .33** | -.03 | .00 | .10 | -.07 | -.04 | .00 | -.04 | -.01 | .09 | -.11 |  |  |  |  |  |  |  |
|  | 15 Extraversion | .12 | -.25** | .07 | .06 | .01 | .17** | .04 | -.11 | -.03 | .09 | -.06 | -.09 | .26** | -.15* |  |  |  |  |  |  |
|  | 16 Agreeableness | .07 | -.10 | .04 | -.07 | -.09 | .09 | .08 | .04 | .01 | .04 | -.03 | -.02 | .20** | -.06 | .05 |  |  |  |  |  |
|  | 17 Conscientiousness | .06 | -.33** | .20** | .19** | .00 | .15* | .16* | .08 | .01 | -.05 | -.11 | .02 | .35** | .06 | .25** | .17* |  |  |  |  |
|  | 18 Openness | .14* | .05 | .13 | .00 | .11 | .11 | .07 | -.05 | .02 | .12 | .04 | -.01 | .05 | .05 | .16* | .23** | .21** |  |  |  |
|  | 19 Disintegration | .25** | .38** | .04 | -.13 | .08 | -.10 | -.03 | .08 | .07 | .03 | .04 | .18* | -.42** | .24** | -.36** | -.14* | -.31** | -.07 |  |  |
|  | 20 REI-R | .07 | -.07 | .05 | .10 | -.01 | .16* | .05 | .12 | -.05 | -.05 | .11 | -.07 | .13 | .01 | .19** | .05 | .31** | .35** | -.03 |  |
|  | 21 REI-E | .23** | .12 | .13 | -.04 | .11 | -.04 | .12 | -.03 | .15* | .11 | -.05 | .03 | -.04 | .01 | .15* | .02 | .14* | .19** | .24** | .17* |
|  | 22 CRT | -.20** | .05 | -.15* | .07 | .02 | .06 | .02 | .03 | -.17* | -.06 | .00 | -.06 | -.06 | -.05 | -.09 | .07 | -.02 | .14* | -.05 | .30** |
| Proximal psychological predictors | 23 Religiosity | .38** | .01 | .04 | .04 | -.15* | .14* | .08 | .04 | .07 | .14* | .06 | .04 | -.03 | .19** | .19** | .12 | .03 | .03 | .17* | .01 |
|  | 24 Spirituality | .23** | .08 | .05 | -.15* | .06 | .01 | .00 | .01 | .12 | .19** | -.05 | -.05 | -.05 | .07 | .04 | .06 | -.02 | .09 | .18** | .04 |
|  | 25 Trust in health professionals | -.10 | -.30** | .09 | .06 | -.02 | .12 | -.12 | .15* | .06 | .03 | -.09 | -.09 | .21** | .10 | .12 | .15* | .15* | .04 | -.17** | .12 |
|  | 26 Mistrust in healthcare system | .21** | .30** | .00 | -.04 | .03 | .10 | .08 | -.13* | -.05 | .03 | .10 | .03 | -.22** | .03 | -.05 | -.14* | -.15* | .00 | .24** | .03 |
|  | 27 Trust in science | -.15* | -.19** | -.07 | .05 | .06 | .16* | -.03 | .10 | .02 | .01 | .02 | -.05 | .14* | -.02 | -.01 | .10 | .22** | .24** | -.20** | .25** |
|  | 28 Political orientation | .10 | .07 | -.12 | .03 | -.15 | -.03 | .02 | .08 | -.12 | -.14* | .09 | .02 | .01 | -.10 | .10 | -.10 | -.15* | -.15* | .03 | -.14* |
|  | 29 GABS | .19** | .21** | .15* | -.02 | .12 | .11 | .10 | .02 | .03 | .04 | .01 | -.05 | -.38** | .42** | -.19** | -.09 | -.04 | .09 | .31** | .10 |
|  | 30 Superstition | .38** | .28** | .11 | -.09 | .06 | .02 | -.08 | .01 | .10 | .07 | .00 | .03 | -.25** | .27** | -.07 | -.06 | -.13 | -.05 | .33** | -.06 |
|  | 31 Conspiracy | .20** | .22** | .03 | .02 | .06 | .04 | .07 | -.02 | .00 | .09 | .08 | .08 | -.10 | .05 | -.03 | -.05 | .02 | .12 | .22** | .09 |
|  | 32 Medical conspiracy | .32** | .29** | .04 | .02 | -.02 | -.07 | .13 | -.05 | .01 | .03 | .08 | .07 | -.13 | .03 | .02 | -.11 | -.05 | -.06 | .31** | .01 |
|  | 33 Magical health beliefs | .40** | .23** | .02 | .03 | -.05 | .14* | .02 | -.09 | -.05 | -.02 | .10 | .03 | -.01 | .03 | .16* | .06 | -.02 | .12 | .21** | .00 |
|  | 34 Doublethink | .16* | .09 | -0.10 | .11 | .08 | -.03 | .01 | .13 | -.03 | .05 | .15* | .05 | -.07 | .08 | .11 | -.09 | -.04 | -.12 | .18** | .12 |
|  | 35 Overconfidence on CRT | .23** | -.07 | .09 | -.12 | -.03 | -.04 | -.02 | -.04 | .17** | .08 | .03 | .09 | .10 | .01 | .09 | .00 | .06 | -.14* | .06 | -.27** |
|  | 36 Naturalness bias | .18** | .10 | .02 | .08 | -.08 | .00 | -.04 | -.01 | .02 | -.02 | -.05 | -.01 | -.06 | -.01 | .07 | .01 | -.09 | -.03 | .10 | -.03 |
|  | 37 Commitment bias | .15* | .04 | .02 | .04 | -.07 | -.09 | .05 | -.01 | .07 | .11 | -.03 | -.01 | .01 | .05 | .12 | -.01 | -.02 | -.15* | .09 | -.04 |
|  | 38 Illusory correlations | .13 | -.03 | -.02 | .02 | -.05 | .02 | .01 | .10 | .18** | .06 | .06 | .15* | .02 | .12 | -.03 | .00 | -.04 | .05 | .16* | -.02 |
|  | 39 Belief bias | .12 | -.02 | -.06 | -.05 | .02 | -.15* | .03 | .01 | .06 | .10 | -.04 | -.05 | -.11 | -.10 | .10 | -.05 | -.04 | .01 | .17* | -.11 |
|  | 40 Probability bias | .16* | .11 | .07 | -.01 | .03 | -.01 | -.01 | .01 | .05 | .02 | -.08 | -.02 | -.17* | .11 | -.08 | .02 | -.10 | -.02 | .14* | -.04 |
|  | 41 Apophenia | .21** | .12 | .02 | .01 | .05 | -.04 | .10 | -.01 | .19** | .12 | .05 | .09 | .07 | .04 | .09 | .03 | -.03 | -.07 | .13 | -.03 |

Table S3 Intercorrelations between all variables in the study (Part 2)

|  |  | 21 | 22 | 23 | 24 | 25 | 26 | 27 | 28 | 29 | 30 | 31 | 32 | 33 | 34 | 35 | 36 | 37 | 38 | 39 | 40 |
| --- | --- | --- | --- | --- | --- | --- | --- | --- | --- | --- | --- | --- | --- | --- | --- | --- | --- | --- | --- | --- | --- |
| DV | 1 TCAM Retrospective |  |  |  |  |  |  |  |  |  |  |  |  |  |  |  |  |  |  |  |  |
|  | 2 iNAR Retrospective |  |  |  |  |  |  |  |  |  |  |  |  |  |  |  |  |  |  |  |  |
| Socio-demographics | 3 Gender |  |  |  |  |  |  |  |  |  |  |  |  |  |  |  |  |  |  |  |  |
|  | 4 Age |  |  |  |  |  |  |  |  |  |  |  |  |  |  |  |  |  |  |  |  |
|  | 5 Urban/Rural |  |  |  |  |  |  |  |  |  |  |  |  |  |  |  |  |  |  |  |  |
|  | 6 Education |  |  |  |  |  |  |  |  |  |  |  |  |  |  |  |  |  |  |  |  |
|  | 7 Marital status |  |  |  |  |  |  |  |  |  |  |  |  |  |  |  |  |  |  |  |  |
|  | 8 Income |  |  |  |  |  |  |  |  |  |  |  |  |  |  |  |  |  |  |  |  |
|  | 9 Household size |  |  |  |  |  |  |  |  |  |  |  |  |  |  |  |  |  |  |  |  |
|  | 10 Children under 18 years |  |  |  |  |  |  |  |  |  |  |  |  |  |  |  |  |  |  |  |  |
|  | 11 BMI |  |  |  |  |  |  |  |  |  |  |  |  |  |  |  |  |  |  |  |  |
|  | 12 Number of chronic conditions |  |  |  |  |  |  |  |  |  |  |  |  |  |  |  |  |  |  |  |  |
| Distal psychological predictors | 13 Honesty-Humility |  |  |  |  |  |  |  |  |  |  |  |  |  |  |  |  |  |  |  |  |
|  | 14 Emotionality |  |  |  |  |  |  |  |  |  |  |  |  |  |  |  |  |  |  |  |  |
|  | 15 Extraversion |  |  |  |  |  |  |  |  |  |  |  |  |  |  |  |  |  |  |  |  |
|  | 16 Agreeableness |  |  |  |  |  |  |  |  |  |  |  |  |  |  |  |  |  |  |  |  |
|  | 17 Conscientiousness |  |  |  |  |  |  |  |  |  |  |  |  |  |  |  |  |  |  |  |  |
|  | 18 Openness |  |  |  |  |  |  |  |  |  |  |  |  |  |  |  |  |  |  |  |  |
|  | 19 Disintegration |  |  |  |  |  |  |  |  |  |  |  |  |  |  |  |  |  |  |  |  |
|  | 20 REI-R |  |  |  |  |  |  |  |  |  |  |  |  |  |  |  |  |  |  |  |  |
|  | 21 REI-E |  |  |  |  |  |  |  |  |  |  |  |  |  |  |  |  |  |  |  |  |
|  | 22 CRT | -.09 |  |  |  |  |  |  |  |  |  |  |  |  |  |  |  |  |  |  |  |
|  |  |  |  |  |  |  |  |  |  |  |  |  |  |  |  |  |  |  |  |  |  |
| Proximal psychological predictors | 23 Religiosity | .13 | -.17* |  |  |  |  |  |  |  |  |  |  |  |  |  |  |  |  |  |  |
|  | 24 Spirituality | .17* | -.07 | .31** |  |  |  |  |  |  |  |  |  |  |  |  |  |  |  |  |  |
|  | 25 Trust in health professionals | -.05 | .12 | .01 | -.04 |  |  |  |  |  |  |  |  |  |  |  |  |  |  |  |  |
|  | 26 Mistrust in healthcare system | .15* | -.04 | .06 | -.01 | -.50** |  |  |  |  |  |  |  |  |  |  |  |  |  |  |  |
|  | 27 Trust in science | -.12 | .24** | -.12 | -.14* | .56** | -.23** |  |  |  |  |  |  |  |  |  |  |  |  |  |  |
|  | 28 Political orientation | .01 | -.16* | .31** | .03 | -.14* | .00 | -.26** |  |  |  |  |  |  |  |  |  |  |  |  |  |
|  | 29 GABS | .03 | .01 | .13 | .11 | .09 | .13* | .03 | -.06 |  |  |  |  |  |  |  |  |  |  |  |  |
|  | 30 Superstition | .13 | -.17** | .32** | .19** | -.11 | .17* | -.25** | .13* | .29** |  |  |  |  |  |  |  |  |  |  |  |
|  | 31 Conspiracy | .16* | -.06 | .14* | .01 | -.19** | .34** | -.11 | -.03 | .24** | .16* |  |  |  |  |  |  |  |  |  |  |
|  | 32 Medical conspiracy | .27** | -.27** | .27** | .15* | -.42** | .46** | -.39** | .18** | .13 | .36** | .63** |  |  |  |  |  |  |  |  |  |
|  | 33 Magical health beliefs | .28** | -.34** | .37** | .21** | -.16* | .27** | -.20** | .23** | .06 | .30** | .42** | .60** |  |  |  |  |  |  |  |  |
|  | 34 Doublethink | .24** | -.19** | .12 | .06 | -.04 | .24** | -.02 | .06 | .20** | .26** | .25** | .40** | .41** |  |  |  |  |  |  |  |
|  | 35 Overconfidence on CRT | .07 | -.86** | .15* | .05 | -.11 | .09 | -.20** | .11 | -.06 | .15* | .09 | .28** | .36** | .26** |  |  |  |  |  |  |
|  | 36 Naturalness bias | .07 | -.20** | .25** | .03 | -.10 | .13 | -.22** | .06 | .07 | .16* | .11 | .26** | .31** | .12 | .17** |  | . |  |  |  |
|  | 37 Commitment bias | .07 | -.16* | .22** | .07 | -.01 | .09 | -.27** | .26** | -.01 | .23** | .20** | .34** | .20** | .17* | .20** | .09 |  |  |  |  |
|  | 38 Illusory correlations | .06 | -.25** | .28** | .04 | .16* | .06 | .08 | .08 | .11 | .18** | .12 | .17* | .21** | .18** | .22** | .04 | .11 |  |  |  |
|  | 39 Belief bias | .27** | -.28** | .09 | .08 | .01 | .03 | -.11 | .14* | -.01 | .08 | .14* | .18** | .23** | .14* | .27** | .03 | .15* | .18** |  |  |
|  | 40 Probability bias | .14* | -.03 | .12 | .19** | -.07 | .05 | -.10 | -.03 | .15* | .26** | .14* | .14* | .15* | .16* | .01 | .08 | .03 | .08 | .13 |  |
|  | 41 Apophenia | .06 | -.15* | .13 | .09 | .06 | .06 | -.03 | -.02 | .06 | .18** | .09 | .16* | .13 | .14* | .22** | .04 | .09 | .05 | .04 | .06 |

Note. TCAM – Traditional complementary alternative medicine, 22-item scale, iNAR – Intentional non-adherence to medical recommendations, 12-item scale, REI-E - Experiential Thinking Style measured via Rational-Experiential Inventory, REI-R - Rational Thinking Style measured via Rational-Experiential Inventory, CRT - cognitive reflection test, GABS - General Attitude and Belief Scale, * p <.05, ** p <.01.

Table S4. Predicting iNAR and TCAM - assessed daily - by distal psychological variables (bivariate MRCM analysis)

|  | Multi-level random coefficient modeling | | | |
| --- | --- | --- | --- | --- |
|  | iNAR_ESM | | TCAM_ESM | |
| Distal psychological predictors (Model 5) | B | t_(222)_ | B | t_(222)_ |
| Honesty | -.01 | -.93 | -.01 | -.91 |
| Emotionality | -.02 | -1.67 | .02 | 1.43 |
| Extraversion | -.01 | -.88 | .04* | 2.45 |
| Agreeableness | -.01 | -.95 | .02 | 1.83 |
| Conscientiousness | -.03** | -2.8 | -.00 | -.03 |
| Openness | -.01. | -.61 | .02 | 1.43 |
| Disintegration | .04** | 3.23 | .04* | 2.39 |
| REI-Rationality | -.02 | -1.9 | -.01 | -1.09 |
| REI-Experientiality | -.00 | -.28 | .04** | 3.13 |
| CRT | -.05** | -3.08 | -.11** | -4.57 |

Table S5. Predicting iNAR and TCAM - assessed daily and retrospectively - by proximal psychological variables (bivariate MRCM analysis)

|  | Multi-level random coefficient modeling | | | |
| --- | --- | --- | --- | --- |
|  | iNAR_ESM | | TCAM_ESM | |
| Proximal psychological predictors (Model 6) | B | t_(213)_ | B | t_(213)_ |
| Religiosity | .00 | 1.63 | .04** | 5.60 |
| Spirituality | .01 | 1.84 | .02** | 2.65 |
| Trust in health professionals | -.01* | -2.14 | -.01 | -.79 |
| Mistrust in the healthcare system | .01 | .49 | .01 | 1.32 |
| Trust in science | -.02* | -2.36 | -.02 | -1.67 |
| Political orientation | .01 | 1.45 | .01 | 1.62 |
| GABS | -.00 | -.26 | .01 | .35 |
| Superstition | .02** | 3.14 | .05** | 4.26 |
| Conspiracy | .00 | .37 | .02 | 1.09 |
| Medical conspiracy | .02** | 3.29 | .03** | 3.45 |
| Magical health beliefs | .02** | 2.87 | .08** | 5.92 |
| Doublethink | .00 | .96 | .00 | .80 |
| Overconfidence on CRT (%) | .00** | 2.88 | .00** | 3.95 |
| Naturalness bias | .02 | .93 | .09** | 3.26 |
| Commitment bias | .01 | 1.08 | .05* | 2.21 |
| Illusory correlations | .04* | 2.09 | .08* | 2.34 |
| Belief bias | .03 | 1.27 | .14** | 3.67 |
| Probability bias | .03 | 1.26 | .05 | 1.09 |
| Apophenia | .00 | 1.31 | .00** | 2.84 |
